# Characterization and pharmacological potential of *Lactobacillus sakei* 1I1 isolated from fresh water fish *Zacco koreanus*

**DOI:** 10.1186/s40199-016-0147-8

**Published:** 2016-03-15

**Authors:** Vivek K. Bajpai, Jeong-Ho Han, Gyeong-Jun Nam, Rajib Majumder, Chanseo Park, Jeongheui Lim, Woon Kee Paek, Irfan A. Rather, Yong-Ha Park

**Affiliations:** Department of Applied Microbiology and Biotechnology, Microbiome Laboratory, Yeungnam University, Gyeongsan, Gyeongbuk 712-749 Republic of Korea; National Science Museum, Ministry of Science, ICT and Future Planning, Daejeon, 32143 Republic of Korea

**Keywords:** Fish microbiota, Lactic acid bacteria, α-glucosidase inhibitory activity, Anti-viral activity, Anti-tyrosinase activity

## Abstract

**Background:**

There are still a large variety of microorganisms among aquatic animals which have not been explored for their pharmacological potential. Hence, present study was aimed to isolate and characterize a potent lactic acid bacterium from fresh water fish sample *Zacco koreanus*, and to confirm its pharmacological potential.

**Methods:**

Isolation of lactic acid bacteria (LAB) from fresh water fish samples was done using serial dilution method. Biochemical identification and molecular characterization of selected LAB isolate 1I1, based on its potent antimicrobial efficacy, was accomplished using API kit and 16S rRNA gene sequencing analysis. Further, 1I1 was assessed for α-glucosidase and tyrosinase inhibitory potential as well as antiviral efficacy against highly pathogenic human influenza virus H1N1 using MDCK cell line in terms of its pharmacological potential.

**Results:**

Here, we first time report isolation as well as biochemical and molecular characterization of a lactic acid bacterium *Lactobacillus sakei* 1I1 isolated from the intestine of a fresh water fish *Z. koreanus*. As a result, *L. sakei* 1I1 exhibited potent antimicrobial effect in vitro, and diameter of zones of inhibition of 1I1 against the tested pathogens was found in the range of 13.32 ± 0.51 to 23.16 ± 0.32 mm. Also *L. sakei* 1I1 at 100 mg/ml exhibited significant (*p* < 0.05) α–glucosidase and tyrosinase inhibitory activities by 60.69 and 72.59 %, in terms of its anti-diabetic and anti-melanogenic potential, respectively. Moreover, *L. sakei* 1I1 displayed profound anti-cytopathic effect on MDCK cell line when treated with its ethanol extract (100 mg/ml), confirming its potent anti-viral efficacy against H1N1 influenza virus.

**Conclusions:**

These findings reinforce the suggestions that *L. sakei* 1I1 isolated from the intestine of fresh water fish *Z. koreanus* might be a candidate of choice for using in pharmacological preparations as an effective drug.

## Background

Microflora of the intestinal tract is an integral part of the whole living organism. A huge number of endogenous and exogenous factors influence determination of composition of microbe populations and affect physiological and biochemical features of the microorganisms. Lactic acid bacteria (LAB) are widely distributed in the intestinal tract of various animals [1], and some of them have played an important role in beneficial functions for industrial animals as probiotics [2]. There have been several reports on LAB occurring among the major microbial populations in animal intestine [2, 3]. It is well established that some LAB improve the intestinal microflora and promote the growth and health of animals [2].

LAB are characterized as Gram-positive, usually non-motile, non-sporulating bacteria that produce lactic acid as a major or sole product of fermentative metabolism which have been classified based on their morphology, physiology and molecular characteristics [4]. Although LAB from food and their current taxonomical status have been reviewed previously [5], taxonomic studies on LAB from animal origin are rare [5]. Moreover, LAB exhibit various medicinal and pharmacological properties against number of microorganisms, including food spoilage and pathogenic bacteria as well as variety of viruses [6].

Consequences of diabetes mellitus type 2 stage are associated with postprandial hyperglycemia due to imbalanced acute secretion of insulin after food intake [7]. Digestive enzymes such as glucosidases are well-known for their ability to break down the larger carbohydrate molecules into simple monosaccharide molecules. Excess production of monosaccharide and less uptake of sugars by the body may result in the development of diabetic complications. Inhibitors of glucosidase enzymes have potent ability to delay the absorption of carbohydrates and reduce the digestion rate of carbohydrates into simple monosaccharides. These features of glucosidase inhibitors allow them to act as anti-diabetic substances because they reduce postprandial blood glucose level thereby preventing the incidences of type-2 diabetes [8].

Tyrosinase, a copper-containing polyphenol oxidase, plays a highly critical role in forming melanin pigments [9]. Previous reports have shown that tyrosinase might also be involved in neuromelanin production and be associated with Parkinson’s disease [10]. Therefore, inhibiting tyrosinase activity is applicable to skin-lightening and in preventing neurodegeneration [11]. Although a broad spectrum of tyrosinase inhibitors are available [12], there is a still need to explore the microbial world for inventing more effective classes of tyrosinase inhibitors from LAB due to their Generally Recognized as Safe “GRAS” status.

Influenza A (H1N1) is the sub-type of influenza A virus that is known as the most common cause of human influenza. Consequences of influenza viruses result in the development of a contagious respiratory disease influenza, also called flu. It has been confirmed that every year over 220,000 hospitalizations and approximately 36,000 annual deaths are reported by influenza viruses in the USA [13]. Current scenario on emergence of life threatening viruses has resulted in enormous attention on finding anti-viral drugs of natural origin due to less potential of currently available anti-viral vaccines [14], in addition to their limited applications [15] against influenza viruses. This has resulted in the increasing need on the development of un-conventional measurements against influenza viruses.

A number of reports have confirmed that LAB are normal flora in gastrointestinal tract of healthy animals like mammals and aquaculture animals including fish [16] with no harmful effects [17]. Probiotics improve intestinal microflora with health beneficial efficacy protecting them against infections by stimulating the immune system, as well as alleviate lactose intolerance, reduce blood cholesterol levels, improve weight gain and feed conversion ratio [18–21]. Isolation of LAB from variety of samples has raised debate over the safety of probiotic bacteria and whether or not the bacteria are actually infectious [22]. Fish viscera are not only rich in different biomolecules but are also rich in beneficial LAB with probiotics properties [23]. Since LAB are reported to be very effective in recovery of biomolecules from fish industry waste [24–26], isolation of LAB from fish industry waste itself becomes all the more important.

Against this background, the main objective of present study was to isolate, and characterize a native LAB strain *L. sakei* 1I1 from intestinal microbiota of fresh water fish *Zacco koreanus*, and to confirm its various pharmacological properties such as antimicrobial, α-glucosidase inhibitory effect, tyrosinase inhibitory effect and antiviral properties against influenza virus H1N1 in terms of its bio-preservative, anti-diabetic, anti-melonogenic and anti-viral potential in order to explore LAB for simultaneous recovery of bioactive compounds of pharmacological significance.

## Methods

### Media and reagents

The Bromocresol Purple (BCP) agar medium was purchased from Sigma-Aldrich (Sigma, MO, USA). The de Man, Rogosa and Sharpe (MRS) agar medium was purchased from Difco (USA). Kojic acid, acarbose, yeast α-glucosidase, p-nitrophenyl-α-D-glucopyranoside, mushroom tyrosinase, and 3,4-dihydroxy-L-phenylalanine (DOPA) were obtained from Sigma (MO, USA). Other chemicals and reagents used were of very pure and high analytical grade.

### Microbial strains

The following microorganisms as pathogenic bacteria were used in this study for preliminary screening including *Staphylococcus aureus* (KCTC 1621), *Escherichia coli* O157:H7, *Salmonella enterica* ATCC 4731, *Bacillus subtilis* KCTC 1021, and *Listeria monocytogenes* KCTC 3569. The bacterial pathogens were obtained from the American Type Culture Collection and Korean Type Culture Collection, respectively and maintained on nutrient agar (NA) medium at 4 °C.

### Collection and sampling of fish samples

A total of 64 fresh water fish samples, belonging to different species were collected from five different rivers and different locations in Korea, supplied by Daejeon National Science Museum, Daejeon. Fish sampling was conducted in the five major river watersheds of Korea (34–42°N, 124–130°E): the Han River, the Nakdong river, the Geum river, the Yeongsan river, and the Sumjin river watersheds during the year 2014. In total, 16 sites consisting of first- through fourth-order streams [27] were sampled for the five major river watersheds; 3 sites in the Han river, 4 sites in the Nakdong river, 3 sites in the Geum river, 3 sites in the Yeongsan river and 3 sites in the Sumjin river. The sampling approach was followed by a modified protocol of the Ohio environmental protection agency (EPA) method [28]. Sampling gears used were casting nets (mesh size, 7 × 7 mm; 1.5 m × 1.5 m × 3.14 m) and kick nets (mesh size, 4 × 4 mm, 1.8 m × 0.9 m), the most common sampling gears used for wading streams. Casting net was applied to habitats with unobstructed open water, viz. riffles, pools, and slow runs, and kick net was used in sites subject to fast current regime and with obstructions, where it is difficult to use a casting net. All sampling procedures and/or experimental manipulations were reviewed following catch per unit effort (CPUE) methods [29], and the collected samples were transported in ice-packed boxes to the Microbiome laboratory, Yeungnam University and stored at −20 °C for further analysis. In addition, this study did not involve any endangered or protected species, hence no specific permissions and ethics were required to collect the fish samples. However, national ethical approval was obtained for fish samples on “Animal Care and Use” by the ethical committee of Daejeon National Science Museum, Daejeon, Korea. All fish samples were of different feeding nature such as insectivore, omnivore, herbivore, and carnivore. Taxonomic identification of the fish species was conducted by the fish expert at the National Science Museum of Korea according to the methods of species identification [30]. A detailed description on variety of fish samples has been given in Table 1.

**Table 1:** Isolation of lactic acid bacteria (LAB) from fresh water fish sample collected from different locations in Korea

Fish samples	Number of LAB isolates		
	Stomach	Intestine	Gill
*Tridentiger bifasciatus*	–	–	–
*Acanthogobius flavimanus*	–	–	1
*Tribolodon hakonensis*	3	–	–
*Pseudobagrus koreanus*	–	–	–
*Coreoleuciscus splendidus*	13	–	–
*Plecoglossus altivelis*	–	1	3
*Misgurnus anguillicaudatus*	1	4	–
*Carassius auratus*	1	1	–
*Pseudorasbora parva*	–	–	–
*Zacco platypus*	1	5	3
*Rhinogobius giurinus*	–	–	–
*Zacco koreanus*	–	–	–
*Zacco temminckii*	–	–	1
*Tridentiger obscurus*	2	–	–
*Zacco koreanus*	1	2	3
*Odontobutis platycephala*	2	2	1
*Rhynchocypris oxycephalus*	3	1	1
*Zacco koreanus*	1	3	2
*Zacco temminckii*	2	2	2
*Rhynchocypris oxycephalus*	3	3	2
*Squalidus gracilis*	5	5	2
*Microphysogobio yaluensis*	4	3	1
*Hemibarbus longirostris*	2	4	2
*Zacco platypus*	2	–	2
*Odontobutis interrupta*	1	2	2
*Rhinogobius brunneus*	1	1	1
*Pseudogobio esocinus*	2	–	1
*Opsariichthys uncirostris*	2	1	1
*Zacco koreanus*	3	3	4
*Rhynchocypris oxycephalus*	1	1	1
*Odontobutis platycephala*	1	1	1
*Pungtungia herzi*	1	1	2
*Zacco platypus*	2	2	2
*Odontobutis platycephala*	3	5	2
*Zacco koreanus*	3	3	2
*Carassius cuvieri*	2	2	2
*Carassius auratus*	3	2	3
*Micropterus salmoides*	2	3	2
*Hemibarbus longirostris*	1	1	2
*Lepomis macrochirus*	3	2	2
*Pseudogobio esocinus*	3	1	2
*Zacco platypus*	2	1	2
*Squalidus chankaensis*	2	2	2
*Rhynchocypris oxycephalus*	4	1	5
*Microphysogobio yaluensis*	3	2	2
*Rhinogobius brunneus*	3	2	2
*Zacco temminckii*	–	3	3
*Odontobutis platycephala*	2	2	2
*Misgurnus anguillicaudatus*	2	3	–
*Zacco koreanus*	2	3	4
*Hemibarbus labeo*			
*Pungtungia herzi*	2	3	2
*Zacco platypus*	3	2	1
*Microphysogobio yaluensis*	3	2	1
*Odontobutis platycephala*	3	1	1
*Pseudogobio esocinus*	1	3	2
*Squalidus gracilis*	4	–	2
*Zacco temminckii*			
*Microphysogobio yaluensis*	1	–	3
*Squalidus gracilis*			
*Pungtungia herzi*			
*Zacco platypus*	–	2	1
*Pseudogobio esocinus*	–	–	3
*Odontobutis platycephala*	–	–	–
Total	117	99	96

### Isolation, sub-culturing and maintenance of LAB from fish samples

For isolation of lactic acid bacteria (LAB) from fresh water fish samples, a previously developed standard serial dilution method was adopted [31]. In brief, scarification of experimental fish was done in a sterilized clean bench using sterilized knife and forces. To isolate LAB from fresh water fish samples, dissected fish tissues such as stomach, gill and intestine were used since these are known major reservoirs of microbial community in fish. Each fish sample was dissected, and stomach, gill and intestine were collected separately. Each part was weighed and homogenized using a pestle-mortar followed by serial dilution in phosphate buffer saline (PBS) using Bromocresol Purple (BCP) agar medium. Each homogenized sample was put in 1 ml of PBS and vortexed vigorously in order to make a uniform inoculum size followed by its serial dilution to the maximum serial dilution factor from 10^−1^ to 10^−9^. Finally an inoculum of 100 μl was spread on BCP agar plates, and plates were sealed using paraffin and incubated at 37 °C for 24 h. Identification of LAB isolates was based on the clear zone around the colony on BCP agar plates [21]. Each set was prepared in triplicate and positive results were confirmed. Representative colonies were picked from plates and well-isolated colonies were inoculated into fresh MRS broth for stock preparation. For long term storage, stock cultures were maintained at −20 °C in MRS broth. A detail of number of LAB strains isolated from variety of fish samples has been summarized in Table 2.

**Table 2:** Biochemical characterization of *Lactobacillus sakei* (1I1) based on carbohydrate interpretation using API 50 CHL kit

Active ingredient	Result	Active ingredient	Result
Glycerol	–	Salicin	+
Erythritol	–	D-cellobiose	+
D-arabinose	–	D-maltose	+
L-arabinose	+	D-lactose (bovine origin)	+
D-ribose	+	D-melibiose	+
D-xylose	+	D-saccharose	+
L-xylose	–	D-trehalose	+
D-adonitol	–	Inulin	–
Methyl-β-D-xylopyranoside	–	D-melezitose	+
D-galactose	+	D-raffinose	–
D-glucose	+	Amidon (starch)	–
D-fructose	+	Glycogen	–
D-mannose	+	Xylitol	–
L-sorbose	–	Gentiobiose	+
L-rhamnose	+	D-turanose	+
Dulcitol	–	D-lyxose	–
Inositol	–	D-tagatose	+
D-mannitol	+	D-fucose	–
D-sorbitol	+	L-fucose	–
Methyl-α-D-glucopyranoside	–	D-arabitol	–
N-acetylglucosamine	+	Potassium gluconate	+
Amygdalin	+	Potassium 2-ketogluconate	–
Arbutin	+	Potassium 5-ketogluconate	–
Esculin	–		

(−): The bacterium does not use this carbohydrate; (+): The bacterium uses this carbohydrate

### Screening of LAB strains on the basis of anti-pathogenic assay

To confirm the bio-preservative and pharmacological potential of LAB strains isolated from variety of fresh water fish samples, anti-pathogenic assay was performed in vitro using different pathogenic microorganisms including *Staphylococcus aureus* KCTC 1621, *Escherichia coli* O157:H7, *Salmonella enterica* ATCC 4731, *Bacillus subtilis* KCTC 1021, and *Listeria monocytogenes* KCTC 3569. The agar well diffusion method [32] was used for anti-pathogenic assay. Petri plates were prepared by pouring 20 ml of nutrient broth (NB) medium (BD Difco™) and allowed to solidify. Plates were dried, and a 24 h grown culture (200 μl) of each test organism of standardized inoculum suspension (10^7^ CFU/ml) was poured and uniformly spread, and the inoculum was allowed to dry for 5 min. The wells were made by using sterilized borer where 100 μl cell free supernatant of isolated LAB strains was poured in each well against each of the tested pathogen. Negative controls were prepared using the same solvent employed to dissolve the samples. Antibacterial activity was evaluated by measuring the diameter of inhibition zones against the tested bacteria. Each assay in this experiment was replicated three times.

### Morphological and biochemical identification of LAB isolate

Morphological identification of one of the selected isolates 1I1, based on its potential efficacy in anti-pathogenic assay, was conducted by observing colony shape on BCP agar plates, Gram-staining, and cell morphology using microscope. Selected isolate was biochemically identified using API 50CH strips with API 50CHL medium at species level based on the instructions of manufacturer (API 50 CHL, BioMerieux, France). In brief, freshly-grown bacterial colony of the selected LAB isolate was picked-up and inoculated in MRS medium at 36 °C for 24 h, and then the bacterial culture was serially diluted to prepare desired concentration of 10^8^ CFU/ml [33]. From this, aliquot (2 ml) was inoculated into APL 50 CHL medium (10 ml), and mixed by gentle inversion. Then, a bacterial suspension (120 μl) was inoculated into API 50 CH strips that were pre-overlaid with mineral oil followed by further incubation for 48 h before measuring the color change abilities. Finally, strips were processed for analyzing the API profiles using computer APILAB Plus Version.

### Molecular characterization of LAB isolate

Molecular methods are important for bacterial identification [34], and possibly more accurate for LAB than conventional phenotypic methods. In this study, LAB isolate 1I1 showing profound antimicrobial efficacy against pathogenic bacteria was characterized by 16S rRNA gene sequencing analysis. The gene sequences were compared in the National Center for Biotechnology Information (NCBI) for homology using BLAST and multiple-aligned with 16S rRNA gene sequences of different strains for similarity using ClustalW program coupled with MEGA 5. A neighbor-joining method was employed to construct the phylogenic tree using MEGA 5 software.

### Pharmacological evaluation of *L. sakei* 1I1

#### Extraction and sample preparation

Selection of an accurate methodology is highly recommended for better metabolite extraction and high amount of yield recovery. Selective use of solvents has always been recommended for the extraction of specific category of metabolites such as polar and/or non-polar or less polar substances based on the solubility of compounds. Since ethanol can significantly extract majority of biologically active secondary metabolites from variety of microbial (outer and inner environment of bacterial cell) extract samples [35], in this study, ethanol solvent system was selected for extraction purposes. Briefly, to obtain ethanol extract, *L. sakei* 1I1 was grown in MRS broth for 36 h at 37 °C, and then double volume of ethanol was added to the culture broth followed by shaking for 4 h to kill the culture. After that, mixture was centrifuged at 8,000 rpm for 20 min. The upper solution containing ethanol extract was collected, vacuum evaporated and freeze-dried. The yield of ethanol extract of LAB strain *L. sakei* 1I1 was found as 19.34 %. Different test concentrations of freeze-dried ethanol extract of 1I1 were prepared in triple-distilled sterilized water.

#### Determination of α-glucosidase inhibitory activity

It is not known whether LAB colonizing the human gut possess inhibitory potential against digestive enzymes glucosidases. Hence, this study was undertaken to evaluate α-glucosidase inhibitory potential of 1I1 in order to confirm its type II anti-diabetic efficacy according to the chromogenic method with minor modifications as described previously [36]. Briefly, 50 μl of various concentrations (100, 50, 25, 5 and 1 mg/ml) of 1I1 ethanol extract and 100 μl of α-glucosidase dissolved in 0.1 M phosphate buffer (pH 6.9), were mixed in a 96-well microplate and incubated at 25 °C for 10 min. After pre-incubation, 50 μl of p-nitrophenyl-α-D-glucopyranoside (5 mM) in the same buffer (pH 6.9) as a substrate solution was added to each well. The reaction mixture was incubated at 25 °C for 5 min. Absorbance was recorded using a microplate reader (Tecan, Infinite M200, Mannedorf, Switzerland) at 405 nm before and after incubation with p-nitrophenyl-α-D-glucopyranoside solution and compared to that of control, having 50 μl of buffer solution instead of test solution. Acarbose at various concentrations (0.3125, 0.625, 1.25, 2.5 and 5 μg/ml) was used as a standard drug. Experiments were performed in triplicate, and enzyme inhibitory effect of the samples was calculated by the formula:

$$ Inhibition\kern0.5em \left(\%\right)=\left( Control\kern0.5em  absorbance- Sample\kern0.5em  absorbance/ Control\kern0.5em  absorbance\right)\times 100 $$

#### Determination of tyrosinase inhibitory activity

Tyrosinase inhibitory activity of ethanol extract of *L. sakei* 1I1 was measured based on the method reported by Fawole et al. [37] with slight modifications. Briefly, 100 μl of different concentrations (100, 50, 25, 5 and 1 mg/ml) of 1I1 ethanol extract were mixed with 0.175 M sodium phosphate buffer (600 μl) (pH 6.8). After that, 200 μl of L-DOPA solution (10 mM) was added to each well, followed by addition of 200 μl of tyrosinase (110 units/ml in 0.175 M sodium phosphate buffer) into the reaction mixture, and incubated at 37 °C for 2 min. Further, the amount of dopachrome produced in the reaction mixture was measured at 475 nm using an ELISA reader. Kojic acid (15.63, 31.25, 62.5, 125 and 250 μg/ml) was used as a positive control. All the steps in the assay were conducted at room temperature. Experiments were performed in triplicate, and enzyme inhibitory effect of the samples was calculated as follows:

$$ Inhibition\kern0.5em \left(\%\right)=\left( Control\kern0.5em  absorption- Sample\kern0.5em  absorption\right)/ Control\kern0.5em  absorption\times 100 $$

### Determination of antiviral effect

#### Harvesting of H1N1 virus from embryonated egg

A pathogenic H1N1 influenza virus (A/ Korea/01/2009) was procured from Korea Centers for Disease Control and Prevention, South Korea. Propagation of influenza virus (H1N1) was maintained on MDCK cell-line for 72 h at 37 °C, in a carbon dioxide (4 %) environment, and allantoic fluid was stored at −80 °C before use. Harvesting of virus H1N1 was accomplished from infected MDCK cell by centrifugation (1,500 rpm) for 5 min. The H1N1 titer was determined as 10^6.5^ median embryo infection dose (EID_50_)/0.1 ml as reported by Rather et al. and Reed and Muench [6, 38].

#### Assay test against H1N1 virus on MDCK cells

MDCK cell-line was cultured and maintained on DMEM medium with 10 % (v/v) fetal bovine serum (FBS), 1 % (v/v) penicillin (100 U/mol), and streptomycin (100 μg/ml) solution. After filter sterilization, ethanol extract (200 ~ 12.5 mg/ml) of *L. sakei* 1I1 was serially diluted in DMEM solution with FBS (2 %, v/v), 1 % (v/v) penicillin (100 U/mol), and streptomycin (100 μg/ml) solution. The H1N1 influenza virus was treated with a two-fold dilution of test sample of 1I1 in sterilized distilled water at 37 °C, under 5 % carbon dioxide for 1 h. Further, this reaction mixture was injected into MDCK cell-line, followed by incubation in DMEM solution with FBS (2 %, v/v) at 37 °C in a humidified chamber under 5 % carbon dioxide for 48 h. Confirmation of antiviral activity was made by observing the plates after 72 h for cytopathic effect (CPE). No CPE confirmed the presence of antiviral activity [6, 39].

### Statistical analysis

Each experiment was performed in triplicate to calculate mean ± SD, and data were analyzed using one-way ANOVA to find statistical significance at *p* < 0.05.

## Results and discussion

### Isolation of LAB strains from fish samples

As presented in Table 1, a total of 312 LAB strains were isolated on BCP media from different tissues samples such as stomach, intestine and gills of 54 fresh water fish samples. Among three samples utilized for isolation of LAB isolates, stomach tissue showed higher number of LAB isolates (116) followed by intestine (99) and gills (96). Interestingly, 13 LAB strains were isolated from stomach tissue of one of the fish samples *Coreoleuciscus splendidus*. Surprisingly, no LAB strain was isolated from any fish tissue samples (stomach, intestine and gill) of *Tridentiger bifasciatus, Pseudobagrus koreanus, Pseudorasbora parva, Rhinogobius giurinus, Zacco koreanus, Hemibarbus labeo, Zacco temminckii, Squalidus gracilis, Pungtungia herzi* and *Odontobutis platycephala*. In this study, a higher number of LAB were isolated from stomach tissue sample of a fresh water fish *Coreoleuciscus splendidus*. Also one of the fish samples *Zacco platypus* possessed higher number of LAB isolates in its stomach, intestine and gill microbiota as compared to other fish samples (Table 1).

Nair et al. [40] also reported distribution of different genera of LAB in fresh and frozen fish and prawn. They observed that from the cultures, 60 in fresh fish, 65 in fresh prawn and 80 % each in frozen fish and prawn belonged to the *Lactobacillus* genus. It is interesting to note that majority of the *Lactobacillus* spp. that have been isolated from fresh and frozen fish/prawns were those species which were commonly found on meat, animals and humans [41].

### Anti-pathogenic potential of *L. sakei* 1I1

**Fig. 1:**
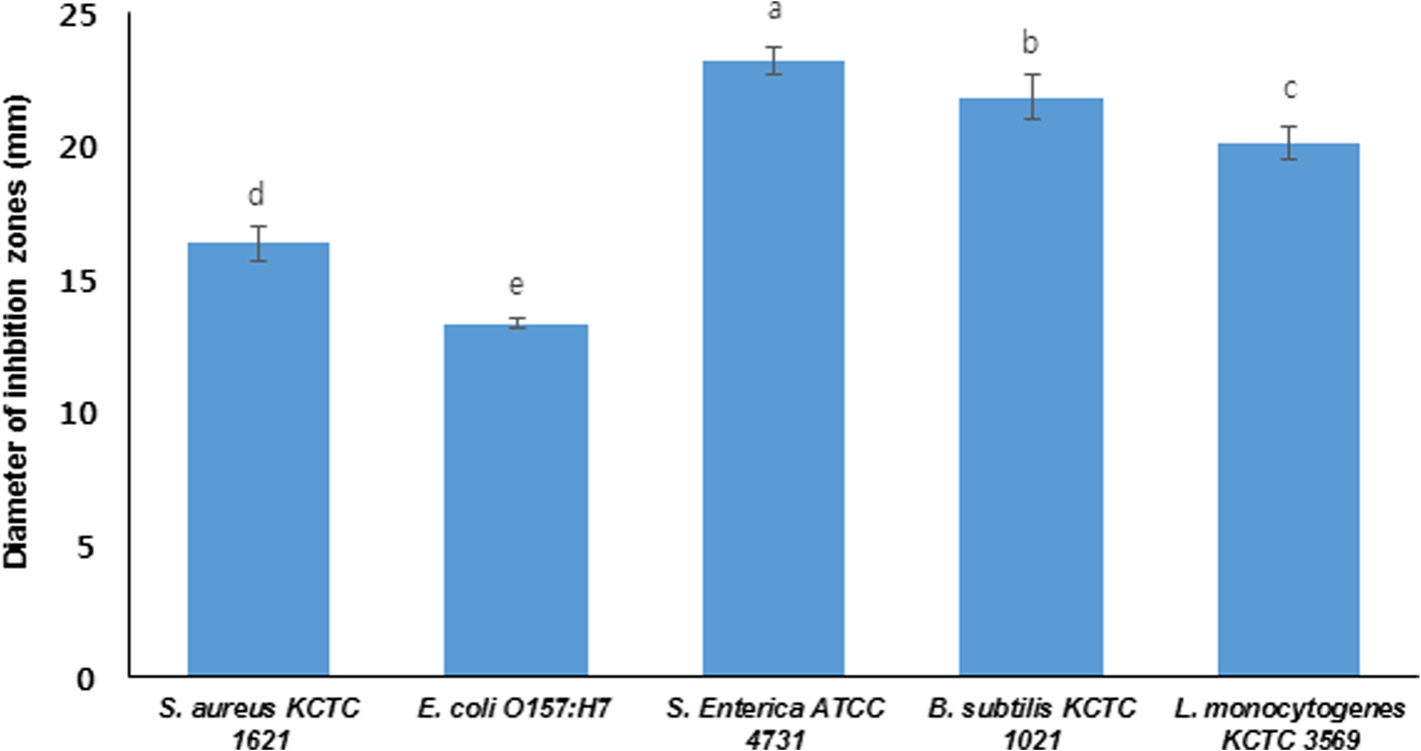
Diameters of inhibition zones of cell free supernatant of *L. sakei* 1I1 against test pathogens in anti-pathogenic assay. Data are expressed as mean ± SD (*n* = 3). Values with different superscripts are significantly different (*p* < 0.05)

All the LAB strains isolated from various parts (stomach, intestine and gill) of fresh water fish samples were named accordingly coded with initial character of their respective tissue origin. Further, each LAB isolate was grown in MRS medium at 37 °C for 24 h. The culture was centrifuged at 12,000 rpm for 15 min to obtain cell free supernatant (CFS). Filter sterilize supernatant was further subjected to evaluate antimicrobial efficacy against pathogenic microorganisms using agar well diffusion assay. In each instance, diameter of inhibition zone was quantified against each bacterial species, and some of the tested strains of LAB showed remarkable antimicrobial activity against the tested pathogenic bacteria (data not shown). It was found in this study that the LAB isolated from the gill and intestine samples exhibited higher anti-pathogenic effect compared to LAB strains isolated from stomach samples. The findings of this study confirmed that the LAB strains isolated from the fresh water fish samples have potent therapeutic and biological potential to develop natural and novel types of antibiotics to combat against pathogenic microorganism causing severe diseases in humans and animals. Based on the result of aforementioned test, one of the strains *Lactobacilli sakei* 1I1, isolated from fresh water fish *Zacco koreanus*, showing highest antimicrobial activity against the tested pathogens (Fig. 1) was chosen as an active LAB isolate and was subjected to biochemical and molecular identification. Further, *L. sakei* 1I1 was assessed for its various pharmacological activities including anti-diabetic, anti-melanogenic and anti-viral efficacy.

### Morphological and biochemical identification

Morphological identification of LAB isolate 1I1 was carried out as per the schemes outlined in the Bergey’s manual of Systematic Bacteriology [42]. Small yellow colonies of similar sizes that appeared on BCP agar using pour-plating method confirmed the presence of *Lactobacillus* strain as also reported previously [43]. The efficiency of detection of other Bifidobacterium such as *B. infantis* on BCP was very low, while *B. bifidum* did not grow on BCP even under anaerobic conditions [43]. Although plate count agar with Bromocresol Purple is a recommended medium for enumeration of LAB from variety of samples, it does not support the differentiation of each LAB in a mixed culture. It is known that BCP agar also prevents formation of colonies by concomitant bacteria and hence widely considered specific medium for the selective enumeration of LAB as also reported by others [44].

Further, biochemical analysis of 1I1 was done by using API50 strip kit and selected strain was identified as a Gram-positive and rod-shaped isolate which was found most closely associated to *L. sakei* (Table 2). The API web software confirmed that strain 1I1 showed typical utilization of carbohydrates that included L-arabinose, D-ribose, D-xylose, D-galactose, D-glucose, D-fructose, D-mannose, L-rhamnose, D-mannitol, D-sorbitol, N-acetylglucosamine, amygdalin, arbutin, salicin, D-cellobiose, D-maltose, D-lactose, D-melibiose, D-saccharose, D-trehalose, D-melezitose, gentiobiose, D-turanose, D-tagatose, and potassium gluconate (Table 2). Color change from violet to yellow in the strip capsule indicated complete fermentation of 1I1. Recently Casaburi et al. [45] also phenotypically and biochemically identified *Lactobacillus* species with the use of API50 kit isolated from fermented sausage.

### Molecular characterization of *L. sakei* 1I1

**Fig. 2:**
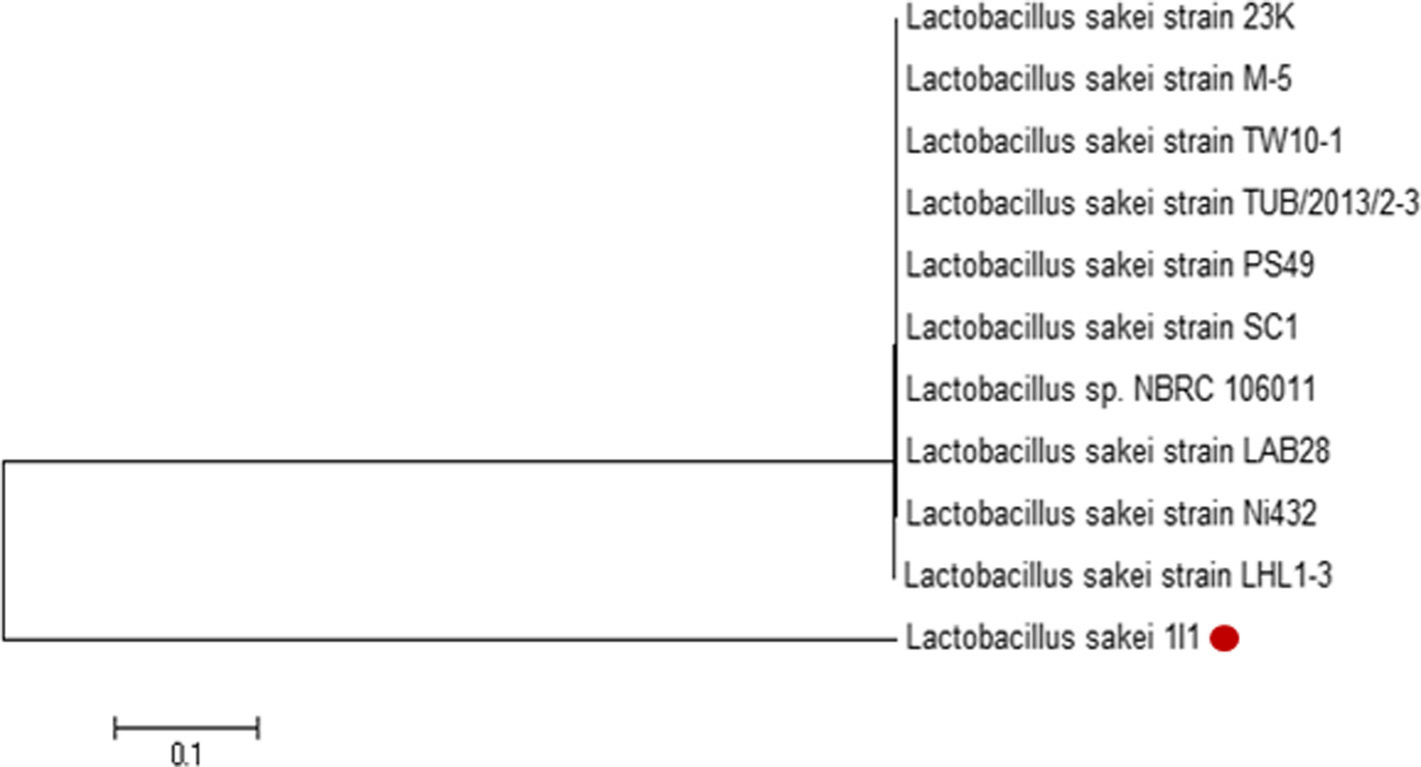
Neighbor-joining phylogenetic tree showing the position of strain *L. sakei* 1I1 among the different *Lactobacillus* strains based on 16 s rDNA sequences

Molecular identification of 1I1 was based on using 16S rRNA gene sequencing analysis. As a result, on the basis of molecular analysis with 16S rDNA gene sequencing, selected strain showed 99.9 % similarity with different *L. sakei* spp. (Fig. 2). The sequence was submitted in GenBank with nucleotide accession number KT372706. Thus, the strain was finally confirmed as *L. sakei* 1I1. Jini et al. [46] also isolated two potential isolates of LAB such as *Enterococcus faecalis* and *Pediococcus acidilactici* from fresh water fish microbiota having anti-pathogenic effect against human pathogens. Zapata [21] studied intestinal microflora of *Oreochromis niloticus* fish to isolate and identify LAB as new probiotics with a possibility to use them in aquaculture. It is known that the microbiota of fish is affected by nutritional, physiological and environmental factors, and it is also expected that the microbial population of fish vary among species [47]. Nevertheless, some authors have considered that seasonal change could be a decisive factor [48], indicating that it is very likely that feeding habits did not have a significant influence on fish LAB composition, when the fish species were grown in the same conditions. Consistent with these findings, our results showed that all species collected in different months had a different LAB composition, represented by various number and variety of LAB species.

### α-Glucosidase inhibitory activity

In this study, anti-diabetic efficacy of *L. sakei* 1I1 was confirmed in α-glucosidase inhibition assay. The α-glucosidase inhibitory activity of ethanol extract of 1I1 was determined using p-nitrophenyl-α-D-glucopyranoside (pNPG) as a substrate and compared with standard compound acarbose. Higher blood glucose levels result in the development of a chronic metabolic disorder called diabetes mellitus. Prevention and control of after-meal blood glucose levels are considered preventive measures to treat diabetes at early stage which may result in the reduction of carbohydrate absorption from food by the inhibitory effect of α-glucosidase-like digestive enzymes [49]. Though commercial drugs as inhibitors of α-glucosidase enzymes are available, an approach on the development of new types of effective alternative measures is needed for inhibiting the action of these digestive enzymes to meet drug cost potential and to reduce the adversary side effects of chemical-based enzyme inhibitors. Moreover, glucosidases may play an active role in the carbohydrate metabolism of beneficial and pathogenic LAB species. Glucosidase activity is widespread among LAB and β-glucosidases release a wide range of plant secondary metabolites from their β-D-glucosylated precursors [50]. Plant metabolite deglycosylation has been shown to improve the flavour or fragrance of fermented products. It also increases the bioavailability of health-promoting, antioxidative plant metabolites [50]. For instance, soybeans contain high concentrations of β-glucosides genistin and daidzin which are hydrolyzed by β-glucosidase activities of LAB during soy milk fermentations. Fermentations with LAB could also increase the concentrations of bioactive isoflavones in traditional oriental herbal medicine formulas [50]. Cassava contains high concentrations of toxic cyanogenic glucoside linamarin, and LAB contribute to linamarin degradation by β-glucosidase activities [50]. In addition, there is considerable interest in the α- and β-glucosidase activities of LAB that conduct the malolactic fermentation of wine. The main volatile constituents of the primary wine aroma are terpenoid compounds derived from the grapes. As these can be released from glycosylated precursors, β-glucosidase activities of malolactic bacteria are of interest due to their impact on the aroma profile of wines [50].

**Fig. 3:**
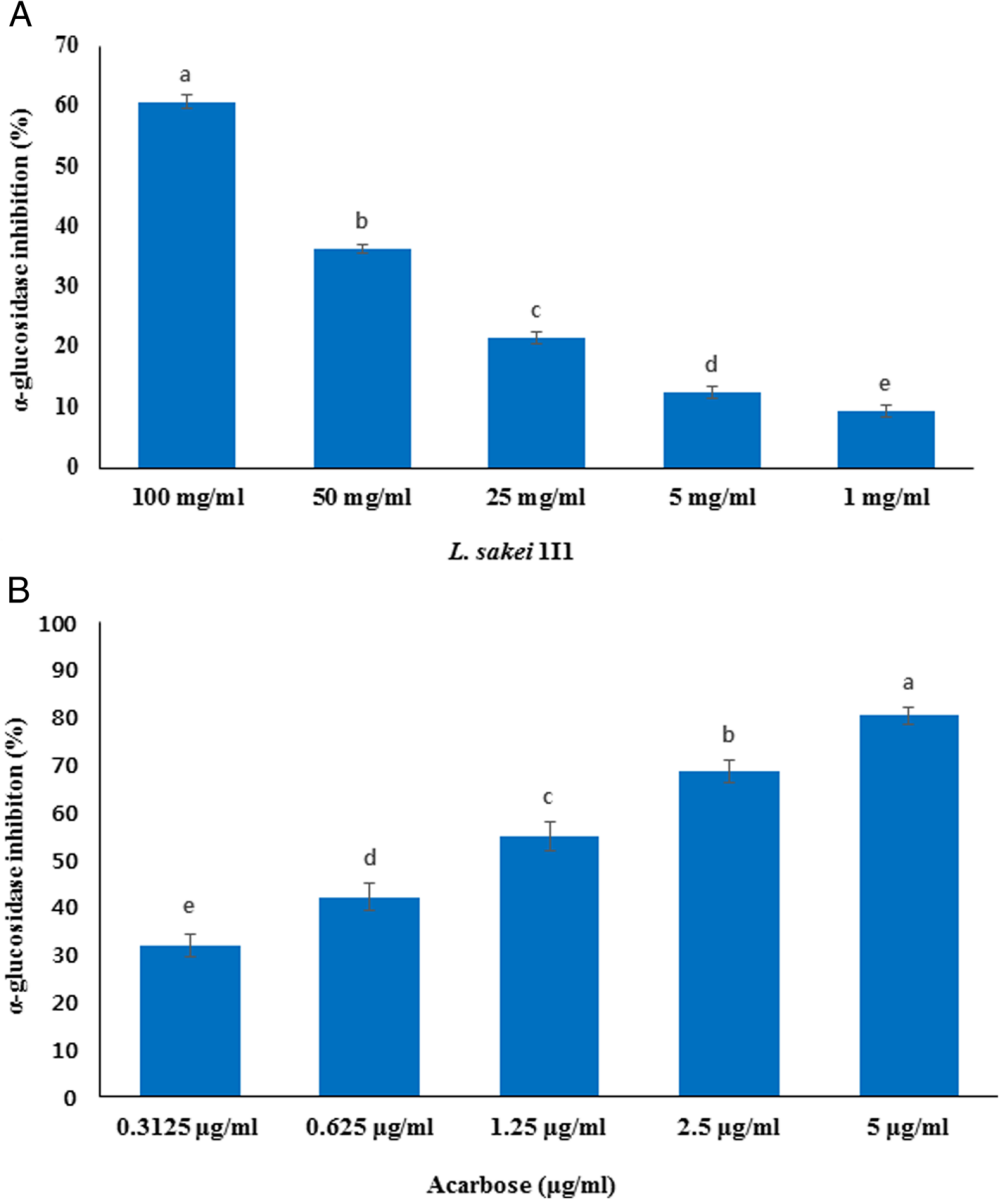
α-glucosidase inhibitory effect of ethanol extract of *L. sakei* 1I1 (**a**) and standard drug compound acarbose (**b**). Data are expressed as mean ± SD (*n* = 3). Values with different superscripts are significantly different (*p* < 0.05)

The α-glucosidase inhibitory activity of ethanol extract of 1I1 was found to be in a concentration-dependent manner (Fig. 3a). The ethanol extract of 1I1 at different concentrations of 1, 5, 25, 50 and 100 mg/ml showed the inhibition of α-glucosidase by 9.34, 12.53, 21.54, 36.32 and 60.69 %, respectively (Fig. 3a). On the other hand, acarbose as a standard drug at various concentrations (0.3125, 0.625, 1.25, 2.5 and 5 μg/ml) displayed α-glucosidase inhibitory activities by 31.84, 42.07, 54.99, 68.59, and 80.32 %, respectively which were also found in a concentration-dependent manner (Fig. 3b).

Similarly, Ramchandran and Shah [51] reported α-glucosidase inhibitory activity of some selected LAB strains, isolated from yogurt starter culture and it was found that all selected strains such as *L. casei*, *L. acidophilus*, *L. delbrueckii* ssp. *bulgaricus*, and *Bifidobacterium longum* exhibited considerable amount of α-glucosidase inhibitory activity, being very high (>80 %). Recently Panwar et al. [52] also reported that LAB strains present in the human gut showed α- and β-glucosidase inhibitory activities as well as reduced blood glucose responses in vivo. Lastly, the inhibitory activity of whole culture ethanol extract of 1I1 observed was clearly in a concentration-dependent, indicating that intracellular cytoplasmic contents or products of bacterial metabolism may be responsible for this activity. Yet this LAB strain, isolated from fish microbiota has not been studied, and it appears that this organism may have some anti-diabetic properties along with its probiotic nature.

### Tyrosinase inhibitory activity

Melanin is a color-determinant naturally found in animals, plants, and microorganisms. Production of various types of melanins is a process of number of enzymatic and non-enzymatic oxidation steps and polymerization reactions. Due to its color reaction and solubility in alkaline medium, it can be divided in two sub-categories eumelanin and pheomelanin [53]. Mechanism of tyrosinase inhibition could be an important factor in the skin whitening and inhibition of browning reaction [53]. During melanin biosynthesis, L-DOPA substrate converts into L-dopaquinone through various enzymatic oxidation processes resulting in the formation of polymerized melanin [53].

**Fig 4:**
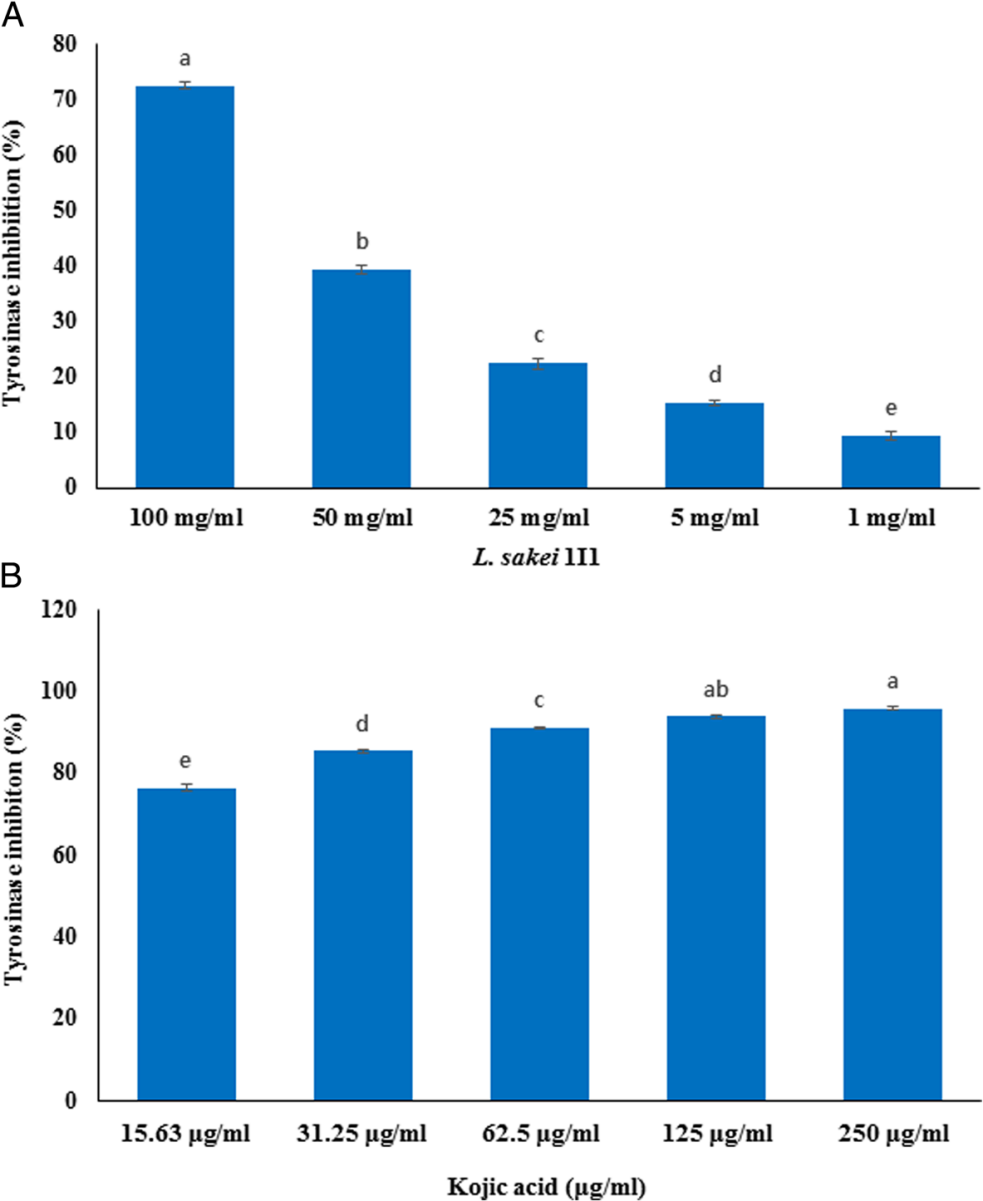
Tyrosinase inhibitory effect of ethanol extract of *L. sakei* 1I1 (**a**) and standard drug compound kojic acid (**b**). Data are expressed as mean ± SD (*n* = 3). Values with different superscripts are significantly different (*p* < 0.05)

The inhibitory effect of *L. sakei* 1I1 ethanol extract on the tyrosinase using a mushroom tyrosinase is demonstrated in Fig. 4. In this assay, ethanol extract of 1I1 (1, 5, 25, 50 and 100 mg/ml) showed inhibition of tyrosinase by 9.36, 15.33, 22.44, 39.34 and 72.59 %, respectively (Fig. 4a). Whereas, mushroom tyrosinase inhibitory effect of kojic acid (15.63, 31.25, 62.5, 125 and 250 μg/ml) was found to be 76.60, 85.52, 91.17, 93.94 and 95.90 %, respectively (Fig. 4b). Kim et al. [54] reported that ethanolic extract of *Cortex radicis* bio-transformed by *Leuconostoc paramesenteroides* PR effectively enhanced the tyrosinase inhibitory activity by 6.5-fold in in vitro*.* Also Usuki et al. [55] demonstrated that LAB and/or their derivatives have wide range of application in the development of skin-whitening and cosmetic products with antioxidant potential that could directly inhibit tyrosinase enzyme activity. Herein this study, ethanol extract of 1I1 also exerted dose-dependent tyrosinase activity. It has been found that inhibitors of tyrosinase play a crucial role to maintain the imbalance of melanin biosynthesis through inhibition of conversion of tyrosine to DOPA, dopaquinone and subsequent formation of melanin [53].

### Antiviral effect against H1N1 influenza virus using MDCK cells

**Fig. 5:**
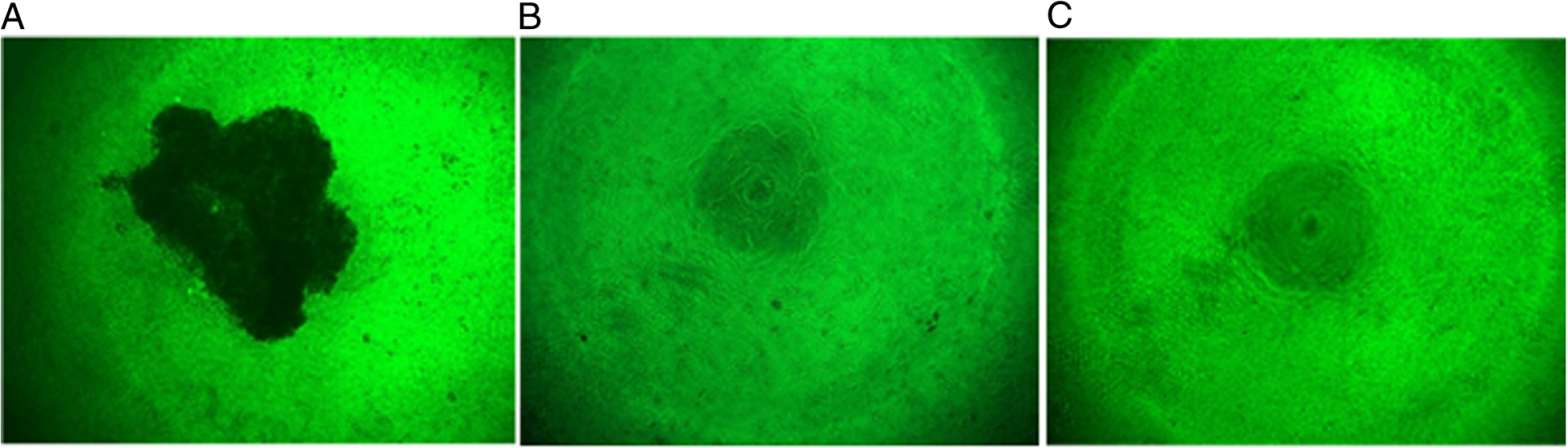
Visualization of cytopathogenic effects of H1N1 virus infection in MDCK cells. Cytopathogenic effect in MDCK cells treated with H1N1 virus (**a**); control MDCK cells without any treatment (**b**); and anti-cytopathic effect in MDCK cells treated with *Lactobacillus sakei* 1I1 and H1N1 (**c**). Pictures were taken under fluorescence microscope at a magnification of 40×

To further confirm the pharmacological potential of 1I1, ethanol extract of 1I1 was tested for its potent antiviral efficacy against H1N1 influenza virus on MDCK cell-line. It was found that H1N1 when used alone caused cytopathic effect (CPE) in MDCK cell-line (Fig. 5a). However, the same CPE was not observed in control MDCK cells (Fig. 5b). Further observations based on microscopic analysis confirmed that MDCK cells treated with H1N1 and ethanol extract of *L. sakei* 1I1 (100 mg/ml) revealed similar morphological pattern as did by the control MDCK cells (no treatment) even after 72 h of the viral injection as shown in Fig. 5c. These findings suggested that 1I1 could be a potential anti-viral candidate to control CPE in MDCK cell-line. Recently we also reported that LAB strain isolated from different sources such a Korean traditional fermented food “Kimchi” exhibited potent antiviral effect against H1N1 influenza virus [6]. Tomosada et al. [56] also observed that it is possible to beneficially modulate the respiratory defense against respiratory syndrome virus (RSV) by using immunobiotics from *Lactobacillus* strains. Oh et al. [57] observed that oral administration of *L. gasseri* might protect a host animal from influenza virus (IFV) infection. Recently Kiso et al. [58] evaluated prophylactic efficacy of *Lactobacillus pentosus* b240 against lethal influenza A (H1N1) virus infection in a mouse model, suggesting it to be an effective candidate in anti-viral therapy.

## Conclusions

This study confirmed that LAB isolate *L. sakei* 1I1, first time isolated from the intestine of fresh water fish *Zacco koreanus* exhibited inhibitory effect on α-glucosidase and tyrosinase enzymes in terms of its potent anti-diabetic and anti-melanogenic activities, respectively as well as exhibited anti-viral effect against influenza virus H1N1 on MDCK cells. These findings reinforce the suggestion that *L. sakei* 1I1 having a broader spectrum of pharmacological activities could be a novel candidate for using in health-care, food system and/or as dietary drug therapies in the treatment of various infectious diseases. Although this study ends with the development of an effective anti-diabetic, anti-melanogenic and anti-influenza candidate *L. sakei* 1I1, in vivo studies using live cultures of 1I1 in animal models should be undertaken to better understand the source of bioactivities.
